# A Rare Case of Hepatocellular Carcinoma With Osteoclast-Like Giant Cells: Favorable Outcome Following Early Surgical Resection

**DOI:** 10.7759/cureus.93435

**Published:** 2025-09-28

**Authors:** Ryohei Aoyama, Kentaro Yasuchika, Masaki Yamada, Naoto Nakamura, Satoshi Yokoyama

**Affiliations:** 1 Surgery, Japanese Red Cross Wakayama Medical Center, Wakayama City, JPN

**Keywords:** hcc with ogcs, hepatocellular carcinoma (hcc), laparoscopic resection, ogc, osteoclast-like giant cells

## Abstract

Hepatocellular carcinoma (HCC) containing osteoclast-like giant cells (OGCs) is an exceptionally rare histological variant, with few cases reported in the literature. These tumors typically exhibit aggressive behavior and a poor prognosis, often presenting at advanced stages. We report the case of a 71-year-old male with early-stage HCC harboring OGCs, incidentally detected during routine surveillance for prior colon cancer. Imaging findings were consistent with conventional HCC, and laparoscopic partial hepatectomy was performed. Histopathological examination revealed well-differentiated HCC with scattered OGCs. The patient recovered uneventfully and has remained recurrence-free for two years postoperatively, as confirmed by routine radiological surveillance. This case highlights the potential for favorable outcomes in HCC with OGCs when diagnosed and treated at an early stage. Further case accumulation is needed to better understand the clinical behavior and optimal management of this rare entity.

## Introduction

Tumors containing osteoclast-like giant cells (OGCs) are rare entities that have been reported in various non-osseous organs, including the ovary, pancreas, urinary tract, thyroid gland, and liver [1]. These multinucleated giant cells resemble osteoclasts morphologically, but their histogenesis remains unclear, with possibilities including reactive transformation or neoplastic origin [2].

In the liver, hepatocellular carcinoma (HCC) is the most prevalent primary malignancy, accounting for approximately 75-85% of all liver cancers globally [3]. HCC is typically suspected based on imaging findings such as arterial phase hyperenhancement and portal venous washout, elevated tumor markers, and underlying risk factors such as chronic liver disease or viral hepatitis. Conventional HCC is the most common histologic subtype and is classified into well, moderately, and poorly differentiated forms based on the degree of cellular differentiation. However, HCC with OGCs is extremely rare, and only a limited number of cases have been reported. This variant may also occur in cirrhotic liver but is typically associated with aggressive behavior, rapid progression, and poor prognosis, often presenting at advanced stages [1,4]. The imaging findings of HCC with OGCs are nonspecific and resemble those of conventional HCC [4]. Due to its rarity, the clinical characteristics, optimal management strategies, and prognostic implications of HCC with OGCs remain poorly defined. Surgical resection is considered one of the treatment options for HCC, but its role in OGC-containing variants has not been well established.

Here, we present a case of HCC with OGCs that was successfully treated with laparoscopic resection, resulting in long-term survival. To our knowledge, this represents only the second documented case of extended survival following surgical intervention for this rare variant, highlighting its potential for curative treatment.

## Case presentation

**Figure 1:**
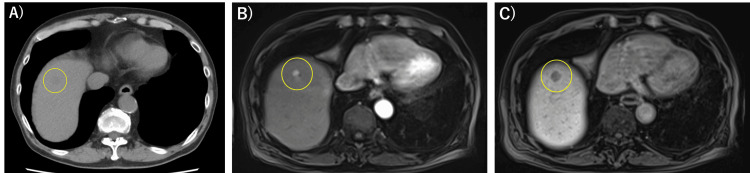
Plain CT and Gd-EOB-DTPA-Enhanced MRI Images (A) Plain CT image: a low-density mass was found in the right lobe of the liver (yellow circle). (B) Gd-EOB-DTPA-enhanced MRI (arterial phase) showing a 2-cm hyperenhancing lesion in segment 8 of the liver (yellow circle). (C) Hepatobiliary phase image demonstrating hypointensity of the same lesion (yellow circle). Gd-EOB-DTPA MRI: gadolinium-ethoxybenzyl-diethylenetriamine pentaacetic acid-enhanced magnetic resonance imaging.

A 71-year-old Japanese man was admitted to our hospital for evaluation and treatment of a liver tumor. He had undergone right hemicolectomy for ascending colon cancer (tub2, pT2N0M0, pStage I) at the age of 69. During routine postoperative surveillance, plain computed tomography (CT) showed a low-density mass in the right hepatic lobe, suggestive of a metastatic lesion from the prior colon cancer (Figure 1A). Contrast-enhanced CT was not performed due to the patient’s known allergy to CT contrast agents.

The patient had achieved a sustained virological response for chronic hepatitis C and was receiving warfarin therapy for atrial fibrillation. He was hemodynamically stable on admission. Laboratory data are summarized in Table 1. Notably, the serum carcinoembryonic antigen (CEA) level was elevated at 10.7 ng/mL (reference < 5.0 ng/mL), and des-γ-carboxy prothrombin (DCP) was markedly elevated at 28,606.7 mAU/mL (reference < 40 mAU/mL). These elevations were suspected to be influenced by the patient’s active smoking status and concurrent warfarin therapy.

**Table 1:** Laboratory Findings on Admission WBC: white blood cell count, Hb: hemoglobin, PLT: platelet count, PT%: prothrombin activity, PT-INR: prothrombin time–international normalized ratio, APTT: activated partial thromboplastin time, Alb: albumin, AST: aspartate aminotransferase, ALT: alanine aminotransferase, T-Bil: total bilirubin, BUN: blood urea nitrogen, Cre: creatinine, AFP: alpha-fetoprotein, CEA: carcinoembryonic antigen, DCP: des-γ-carboxy prothrombin.

Parameter	Value	Reference Range	Unit
WBC	5400	3300–86,000	/μL
Hb	14.7	13.7–16.8	g/dL
PLT	140,000	158,000–348,000	/μL
PT%	25.3	74.4–120	%
PT-INR	2.32		
APTT	33.8	24.1-31.7	sec
Alb	4.1	4.1–5.1	g/dL
AST	30	13–30	U/L
ALT	39	10-42	U/L
T-Bil	0.4	0.4–1.5	mg/dL
BUN	17	8–20	mg/dL
Cre	1.02	0.65–1.07	mg/dL
CRP	0.12	<0.14	mg/dL
AFP	4	<10	ng/mL
CEA	10.7	<5.0	ng/mL
PIVKA-II	28,606.7	<40	mAU/mL

Gadolinium-ethoxybenzyl-diethylenetriamine pentaacetic acid-enhanced magnetic resonance imaging (Gd-EOB-DTPA MRI) revealed a 2 cm lesion in segment 8 of the liver, demonstrating arterial phase hyperenhancement and hepatobiliary phase hypointensity, consistent with typical imaging features of HCC (Figure 1B,C). Based on these imaging findings and his history of HCV infection, the lesion was considered more consistent with a newly developed HCC rather than a hepatic metastasis from previously resected ascending colon adenocarcinoma. Due to the risk of tumor cell seeding associated with needle biopsy, we did not perform a biopsy. Instead, we proceeded with a laparoscopic partial liver resection of segment 8.

**Figure 2:**
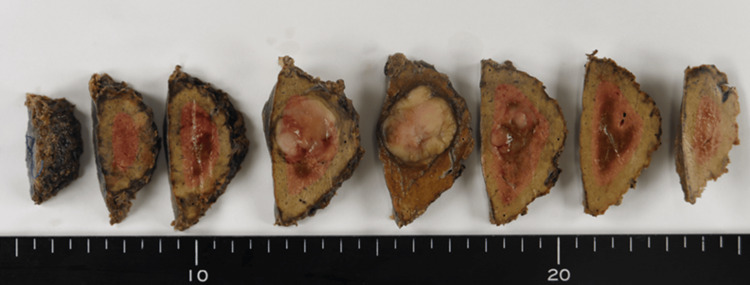
Macroscopic Findings of the Resected Specimen A solid and well-defined tumor 2.2 × 2.0 × 1.8 cm in size was observed.

**Figure 3:**
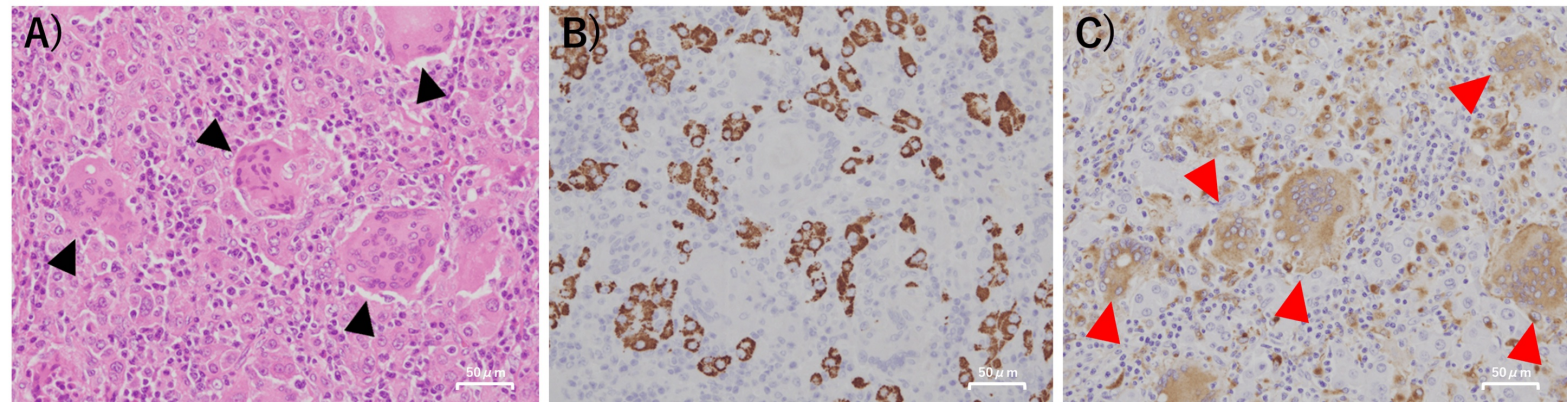
Microscopic Findings of the Resected Specimen (A) Hematoxylin and eosin (H.E) staining (×200) showing trabecular proliferation of HCC cells with numerous OGCs in the background (black arrows). (B) Immunohistochemical staining for HepPar1 demonstrating diffuse positivity in HCC cells (×200). (C) Immunohistochemical staining for CD68 showing positivity in OGCs (×200). Red arrows indicate representative CD68-positive giant cells. HCC: hepatocellular carcinoma, OGC: osteoclast-like giant cell, H.E.: hematoxylin and eosin, HepPar-1: hepatocyte paraffin 1, CD68: cluster of differentiation 68.

The resected specimen demonstrated a solid and well-defined tumor measuring 2.2 × 2.0 × 1.8 cm (Figure 2). Histopathologically, it was diagnosed as well-differentiated HCC. In addition, OGCs were observed in the background (Figure 3A). Immunohistochemical staining demonstrated diffuse HepPar1 positivity in HCC cells and strong CD68 expression in OGCs (Figure 3B,C). Based on these findings, we diagnosed the tumor as a newly developed HCC with OGCs, rather than a metachronous metastasis from the previously resected ascending colon adenocarcinoma. No vascular or bile duct invasion was observed, and surgical margins were negative.

The patient was discharged on postoperative day 9 and has remained recurrence-free for two years, as confirmed by routine radiological surveillance.

## Discussion

HCC is the most common primary liver malignancy, but HCC with OGCs is an extremely rare variant. This tumor type was first reported by Kuwano et al. in 1984 [5].

The imaging findings of HCC with OGCs are not specific and generally show the same radiological features as conventional HCC [4], making radiological differentiation challenging. In the present case, the Gd-EOB-DTPA MRI findings were also suggestive of typical HCC. Therefore, histopathological evaluation remains indispensable for establishing a definitive diagnosis. Although HCC is commonly associated with underlying liver disease, several cases of HCC with OGCs have been reported in patients without prior hepatopathies [6-9], further complicating the diagnostic process.

The treatment approach for this tumor is similar to that of conventional HCC, and surgical resection is an option. However, HCC with OGCs typically exhibits highly aggressive clinical and biological characteristics, leading to a poor prognosis even after surgical resection [1].

The origin of OGCs remains uncertain. Previous reports have proposed that OGCs represent reactive histiocytic cells rather than true malignant components, based on their immunohistochemical profile. Specifically, OGCs consistently express the histiocytic marker CD68 and show a low Ki-67 proliferation index, suggesting limited proliferative activity and a non-neoplastic nature [2,7]. Additionally, OGCs in the liver demonstrate an expression pattern similar to osteoclasts in bone, including markers such as CD68, receptor activator of nuclear factor-kappa B (RANK), and RANK ligand (RANKL). These findings suggest that the histogenesis of OGC formation in liver cancer may follow mechanisms similar to those of bone osteoclastogenesis [2]. To the best of our knowledge, only 11 cases of HCC with OGCs have been reported in the English literature (Table 2) [1,2,4-12].

**Table 2:** Previous Reported Cases of HCC With OGCs in English Literature HCC: hepatocellular carcinoma, OGCs: osteoclast-like giant cells.

No	Author, Ref.	Year	Age (Years)	Sex	Diagnosis	Tumor Size (cm)	Cirrhosis	Treatment	Prognosis
1	Kuwano et al. [5]	1984	54	Male	HCC with OGC	12	Yes	Operation	Death (42 days)
2	Hood et al. [6]	1990	37	Female	Hepatic giant cell carcinoma	-	No	Operation	Death (3 months)
3	McCluggage et al. [10]	1993	71	Male	HCC with OGC	-	Yes	-	Death (1 month)
4	Sasaki et al. [11]	1997	42	Male	Sarcomatoid HCC with OGC	6	Yes	Operation	Death (28 days)
5	Ikeda et al. [2]	2003	76	Male	Sarcomatoid HCC with OGC	-	Yes	Trans-arterial embolization (TAE) + Operation	Death (5 months)
6	Tanahashi et al. [7]	2009	74	Female	Combined HCC and OGC	10	No	Operation	Death (4 months)
7	Lee [12]	2014	64	Male	Sarcomatoid HCC with OGC	6	Yes	Operation	Recurrence (2 months)
8	Dioscoridi et al. [8]	2015	74	Female	HCC with OGC	10	No	Operation	Death (4 months)
9	Dahm [9]	2015	68	Male	Sarcomatoid HCC with OGC	6	No	Operation	Recurrence (3 months)
10	Tsukimoto et al. [1]	2022	70	Male	Recurrent HCC with OGC	2.1	Yes	Operation	No recurrence (a year and a half)
11	Frittoli et al. [4]	2024	77	Male	HCC with OGC	3.8	Yes	Operation	Recurrence (6 months)

Among the patients, eight were male and three were female, with ages ranging from 37 to 77 years (median age, 70 years). Seven patients had underlying hepatopathies, while four did not. The tumors measured between 2.1 and 12 cm in diameter. Sarcomatous transformation of HCC was observed in four cases. Sarcomatous transformation is considered a form of tumor dedifferentiation, and OGCs are occasionally observed in poorly differentiated areas, suggesting a possible association. Surgical resection was performed in 10 patients. However, the prognosis of previous cases was very poor, with 10 patients experiencing recurrence or death within six months after treatment. Only one case has been reported to achieve long-term survival, with a tumor size of 2.1 cm, which was relatively small. Similarly, in our case, the tumor size was also small. These findings suggest that tumor size may be a prognostic factor, underscoring the importance of early detection and timely intervention.

While the presence of OGCs in HCC has been documented in rare cases, their biological and clinical significance remains unclear. This report is limited by the rarity of this histological subtype and the lack of statistically supported data. In our case, the presence of OGCs may represent an incidental histological finding without a clear impact on tumor behavior or prognosis.

## Conclusions

HCC with OGCs is an uncommon histological subtype typically associated with aggressive behavior and poor prognosis. However, this case suggests that early detection and timely surgical intervention can lead to favorable long-term outcomes, highlighting the importance of routine surveillance in at-risk individuals.

Continued documentation and analysis of similar cases are essential to better characterize the clinicopathological features of this variant and to inform future diagnostic and therapeutic strategies.
